# Hidradenitis secondary to nirogacestat, a recently approved desmoid tumor medication

**DOI:** 10.1016/j.jdcr.2024.12.022

**Published:** 2024-12-31

**Authors:** Travis C. Frantz, David Kirwin, Catherine Brahe

**Affiliations:** Department of Dermatology, Navy Medicine Readiness and Training Command San Diego, San Diego, California

**Keywords:** cutaneous drug reaction, desmoid tumor, gamma secretase inhibitor, hidradenitis, hidradenitis suppurativa, HS, nirogacestat

## Introduction

Hidradenitis suppurativa (HS) is a poorly understood chronic inflammatory disorder of the hair follicle that affects apocrine gland-rich and intertriginous areas. HS is a clinical diagnosis characterized by deep seated, painful nodules, frequently in the axilla or groin, that may spontaneously rupture and drain malodorous, purulent, or sanguineous material. Drug-associated HS has been previously reported in the literature implicating new immunomodulating medications such adalimumab, infliximab, vemurafenib, rituximab, and tocilizumab.^1^ However, we present a case of HS secondary to an entirely new class of medication, gamma secretase (GS) inhibitors.

## Case Presentation

We present a case of a 29-year-old man of Latino ancestry with no previous history of chronic skin conditions who presented to our clinic for evaluation of recurrent painful nodules in the groin and the axilla that began 3 months after starting nirogacestat. His past medical history was significant for a diagnosis of a desmoid tumor in his right popliteal fossa at the age of 26 years. Unfortunately, he failed both sorafenib and doxorubicin. The tumor was deemed unresectable due to neurovascular involvement and he was enrolled in a clinical trial of nirogacestat that prohibited the use of concurrent systemic medical therapies.

**Fig 1:**
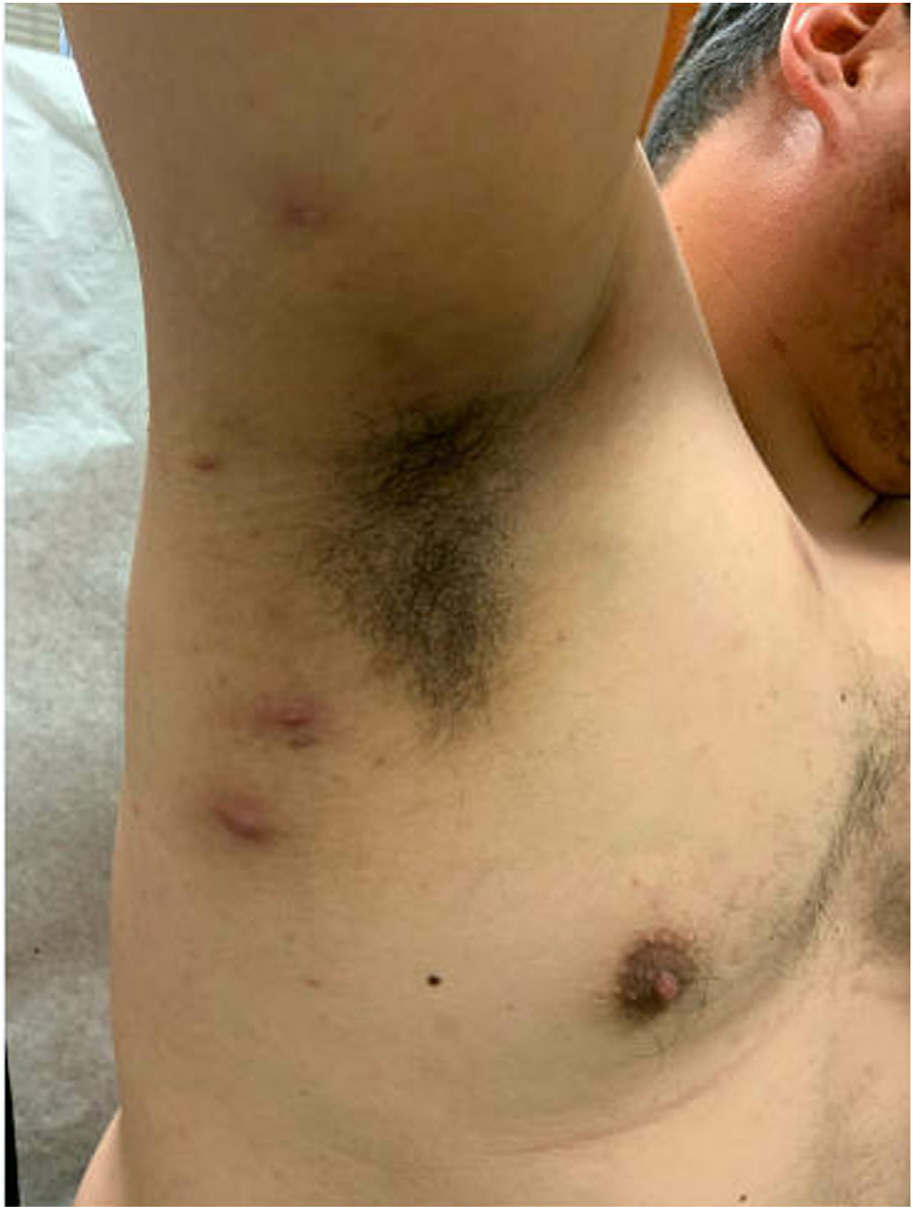
Right axilla demonstrating tender erythematous nodules after 4 months of treatment with nirogacestat at initial evaluation.

**Fig 2:**
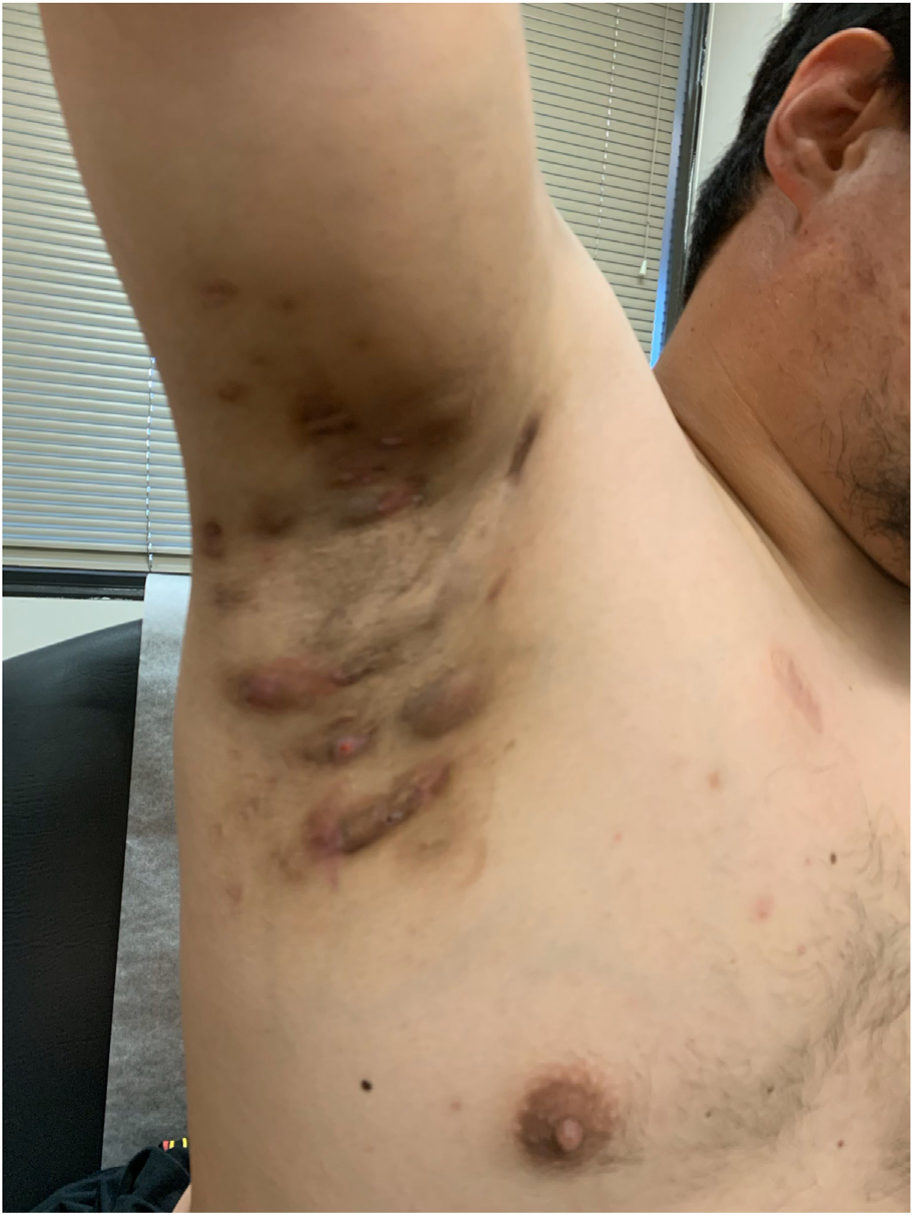
Right axilla demonstrating postinflammatory hyperpigmentation, nodules, scarring, and draining sinus tract after 10 months of treatment with nirogacestat.

On physical examination there were scattered firm tender nodules of the groin and axilla (Fig 1) consistent with Hurley stage 1 HS. Over a 10-month period of treatment with nirogacestat, the patient’s HS continued to progress to Hurley stage 3, demonstrating well-formed sinus tracts, scarring, and draining nodules affecting the axilla (Fig 2) and groin. Due to the clinical trial, the patient’s treatment was limited to laser hair removal, intralesional steroid injections, benzoyl peroxide wash, and topical clindamycin. Surgical deroofing was eventually pursued as the patient’s disease worsened.

## Discussion

Nirogacestat is a GS inhibitor. GS is a multisubunit enzyme complex that performs intramembrane proteolysis on several integral membrane proteins. Genomic and investigational studies have substantiated importance of GS for the maintenance of the pilosebaceous unit.^2^^,^^3^ Multiple authors have demonstrated that loss of function mutations in nicastrin and other subunits of this enzyme are associated with the development of HS.^2^

In a phase 2 study of nirogacestat by Kummar et al,^4^ cutaneous toxicity was reported as common adverse effect. The authors reported a rash without further characterization in 9 of 17 patients.^4^ O’Sullivan Coyne et al^5^ determined that 6 of these patients had follicular and cystic lesions in the intertriginous areas. Additionally, a recent case was published reporting HS as an adverse effect in a patient enrolled in a phase 2 GS inhibitor trial.^6^

Nirogacestat is the first drug approved by the Food and Drug Administration for the treatment of desmoid tumors in November 2023. As an approved medication, medical therapies may be used to treat HS in these patients. Systemic clindamycin and rifampin should be avoided due to interaction with CYP3A4. There are no reported contraindications to biologic therapy. It is important for clinicians to be aware of the serious potential adverse effect of this novel drug.

## Conflicts of interest

None disclosed.
